# Causal relationship between telomere length and risk of intracranial aneurysm: a bidirectional Mendelian randomization study

**DOI:** 10.3389/fneur.2024.1355895

**Published:** 2024-03-11

**Authors:** Bangjie Xu, Jiangbin Ren, Siqi Zhu, Yu Ding, Wei Zhou, Qing Guo, Yan Fang, Jing Zheng

**Affiliations:** ^1^Department of Neurosurgery, The Affiliated Suqian Hospital of Xuzhou Medical University, Suqian, China; ^2^Department of Neurosurgery, The Affiliated Huaian No. 1 People’s Hospital of Nanjing Medical University, Huaian, China; ^3^Department of Oncology, The Affiliated Huaian No. 1 People’s Hospital of Nanjing Medical University, Huaian, China

**Keywords:** telomere length, intracranial aneurysm, Mendelian randomization, causality, single nucleotide polymorphism

## Abstract

**Background:**

Telomere length is closely linked to the aging phenotype, where cellular aging results in the production of a cascade of cell factors and the senescence-associated secretory phenotype (SASP), leading to an inflammatory response. The presence of inflammation plays a crucial role in the formation of intracranial aneurysms. Nevertheless, the relationship between telomere length and intracranial aneurysms remains unclear. This study aims to explore the causal connection between telomere length and intracranial aneurysms through the utilization of Mendelian randomization (MR) analysis.

**Methods:**

Data on telomere length were obtained from the genome-wide association studies conducted on the UK Biobank, comprising a total of 472,174 participants. Data on intracranial aneurysms were obtained from the summary dataset of the Global Genome-wide Association Study (GWAS) conducted by the International Stroke Genetics Consortium. The dataset consisted of 7,495 cases and 71,934 controls, all of European descent. Initially, the linkage disequilibrium score was used to investigate the connection between telomere length and intracranial aneurysms. Subsequently, a bidirectional MR was conducted using two-sample analysis to assess whether there is a causal connection between telomere length and intracranial aneurysm risk. The results were analyzed utilizing five MR methods, with the inverse variance weighted method serving as the main methodology. In addition, we did various analyses to evaluate the presence of heterogeneity, pleiotropy, and sensitivity in the study results. A reverse MR analysis was conducted to investigate potential reverse causal links.

**Results:**

In the forward MR analysis, it was observed that both the inverse variance-weighted and weighted median analyses implied a potential causal relationship between longer telomere length and a decreased incidence of intracranial aneurysms (IVW: OR = 0.66, 95% CI: 0.47–0.92, *p* = 1.49 × 10^−2^). There was no heterogeneity or horizontal pleiotropy. The findings were verified to be robust through the utilization of leave-one-out analysis. The use of reverse MR analysis did not establish a potential causal link between the occurrence of intracranial aneurysms and telomere length.

**Conclusion:**

There may exist a potential correlation between longer telomere length and a decreased likelihood of intracranial aneurysms within the European population. The present study offers novel insights into the correlation between telomere length and intracranial aneurysms. Additional research is required to clarify the underlying mechanisms and validate our discoveries in diverse populations.

## Introduction

Intracranial aneurysms refer to an atypical localized expansion of blood vessels within the brain, typically manifesting as a rupture that results in subarachnoid haemorrhage. This condition is associated with a significant frequency and fatality rate. Currently, the cause of cerebral aneurysms remains uncertain. According to Chalouhi et al. (1), inflammation is suggested to have an essential role in the development of cerebral aneurysms. The initiation of the inflammatory process is triggered by hemodynamic pressures, which subsequently induce extracellular matrix degradation through the action of matrix metalloproteinases (MMPs) and smooth muscle cell death. This progressive weakening of the artery wall ultimately leads to its dilatation and the subsequent development of an aneurysm. The findings of a conducted experimental investigation employing a rat model of arterial aneurysm indicate a potential correlation between increased levels of telomere-binding protein and the development of intracranial aneurysms. This protein inhibits telomerase activity, leading to accelerated telomere shortening (2). Based on current epidemiological research findings, intracranial aneurysms may be directly associated with aortic aneurysms, particularly abdominal aortic aneurysms and thoracic aortic aneurysms. There may be shared genetic and pathological factors between them (3). Recent research findings suggest a potential causal association between the length of telomeres and the occurrence of aortic aneurysms (4). These findings offer promising prospects for the development of tailored treatment strategies. However, the potential existence of a causal association between telomere length and cerebral aneurysms has yet to be established.

Telomere length pertains to the measurement of the repeating heterochromatic area located at the termini of eukaryotic chromosomes. These regions are distinguished by a cap-like structure composed of the nucleotide sequence TTAGGG. Telomere length is a crucial element that influences the process of biological ageing (5). The process of telomere shortening is a natural occurrence that accompanies the ageing process. However, many factors such as inflammation, oxidative stress, and exposure to toxic stimuli can expedite the attrition of telomeres, hence influencing and accelerating the ageing process. When the length of telomeres decreases beyond a specific threshold, cells lose their ability to undergo division and replication, leading to a state known as cellular senescence and apoptosis (6). Cellular senescence is a stress response that can result in a persistent cell cycle stop, notwithstanding the metabolic activity of senescent cells. SASPs, including pro-inflammatory cytokines, growth factors, chemokines, and MMPs, are closely associated with a variety of physiological processes and senescence-related diseases (7). The maintenance of telomere length is contingent upon the collaborative interplay between telomerase and telomere-binding proteins. Telomerase is a ribonucleoprotein complex that plays a crucial role in the extension of telomere sequences. Several meta-analyses (8–10) have indicated a negative association between telomere length and the occurrence of various cardiovascular or cerebrovascular events, including stroke. Moreover, when risk factors such as smoking, obesity, and atherosclerosis are present, the initiation and advancement of intracranial aneurysms correspond to the pattern observed in telomere length. Nevertheless, the connection between intracranial aneurysms and telomere length remains uncertain.

The analysis of MR utilizes genetic variants to evaluate if the observed correlation between an exposure factor and an outcome is in line with a causal relationship. Observational cohorts adhering to traditional methodologies are vulnerable to the influences of confounding variables and the potential for reverse causality effects. In contrast, MR is predicated on the notion of random allocation of genetic variations during meiosis, resulting in a stochastic dispersion of genetic variants among populations. Because these genetic variations are typically unrelated to confounding factors, the differences between carriers and non-carriers can be attributed to differences in the exposure factor (11). This methodology offers substantial empirical support for establishing causal relationships between particular exposures and their corresponding outcomes. The present investigation utilized MR analysis as a methodological approach to elucidate the causal relationship between telomere length and intracranial aneurysms.

## Materials and methods

### Data sources

This study employed MR analysis using GWAS data. All data were sourced from the IEU Open GWAS project or original studies, and all studies obtained ethical approvals and informed participants accordingly. The data pertaining to exposure variables for genetic variants linked with telomere length were acquired from a GWAS meta-analysis with a sample size of 472,174 individuals of European descent (12) (ieu-b-4879). The outcome dataset used in this study consisted of 7,495 cases of intracranial aneurysm and 71,934 controls (Table 1), all of European ancestry (maximum 2% overlap with telomere length GWAS), as shown in Supplementary Table S5. These data were obtained from the International Stroke Genetics Consortium and were derived from a GWAS that focused on intracranial aneurysm (13). The inclusion of individuals of European ancestry was done to reduce the potential influence of ethnic bias. Furthermore, in order to explore the potential existence of reverse causality between telomere length and intracranial aneurysms, a reverse MR study was performed. This research utilized intracranial aneurysms as the exposure variable and telomere length as the outcome variable.

**Table 1:** Characteristics of genome-wide association study (GWAS) data.

Type	Traits	Source	GWAS ID	Ancestry	Sample size
Exposure	TL	UK Biobank	ieu-b-4879	European	472,174
Outcome	IA	Bakker et al.	NA	European	7,495 cases/71,934 controls

TL, telomere length; IA, intracranial aneurysm; NA, not applicable.

### The genetic variations associated with telomere length

First, an extraction of summary data pertaining to single nucleotide polymorphisms associated with telomere length was conducted, utilizing publicly accessible Genome-Wide Association Studies. We picked single nucleotide polymorphisms that showed a significant association with the exposure factor, with a *p*-value less than 5 × 10^–8^. SNP independence was rigorously assessed (*r*^2^ = 0.001, kb = 10,000). Secondly, in accordance with the three fundamental assumptions of MR and employing the PhenoScanner V2 database (14, 15), any single nucleotide polymorphism that exhibits correlation with established confounding factors, including systolic blood pressure, diastolic blood pressure, hypertension, or medications associated with hypertension, is deliberately omitted from the analysis. Instances where instrumental single nucleotide polymorphisms for the exposure variable were absent in the outcome data were omitted from the analysis. Finally, alleles of SNPs across studies were harmonized, palindrome SNPs with unclear allele frequencies were removed, and SNPs located in the MHC region were also excluded.

### LD score regression

The utilization of LD score regression has proven to be a dependable and efficient approach in the identification of the shared genomic architecture that underlies complex characteristics (16). It is primarily based on estimating the heritability of a disease and testing its genetic correlation using complete GWAS data. In the analysis, complete GWAS data for telomere length and intracranial aneurysms were utilized to assess genetic correlation, with a threshold set at *p* < 0.05.

### Mendelian randomization study

In this study, for the horizontal pleiotropy and outliers in MR, an initial detection was conducted using MR-PRESSO. In the event that horizontal pleiotropy was detected, single nucleotide polymorphisms that exhibited a statistical significance level of *p* < 0.05 in relation to this phenomenon were eliminated from the analysis. Furthermore, the MR-Egger regression method was employed to examine the presence of horizontal pleiotropy. When the *p*-value of the MR-Egger intercept is less than 0.05, it suggests the existence of horizontal pleiotropy. After removing outliers and confirming horizontal pleiotropy, MR-PRESSO was applied again. Subsequently, an assessment of the heterogeneity among single nucleotide polymorphisms (SNPs) was conducted utilizing the inverse variance-weighted (IVW) approach and the MR-Egger method. Only SNPs that remained significant after adjusting for horizontal pleiotropy were maintained for further analysis. The Cochran’s *Q* test was utilized to evaluate the presence of heterogeneity in the IVW approach, while the MR-Egger method employed Rucker’s *Q* test for the same purpose. If heterogeneity was statistically significant (*Q* < 0.05) and horizontal pleiotropy was absent, SNPs with MR-PRESSO results *p* < 1.00 were removed, followed by another MR-PRESSO analysis. In the event that MR-PRESSO did not uncover any statistically significant single nucleotide polymorphisms (SNPs), or if the global test in MR-PRESSO had a *p*-value less than 0.05, the radial IVW and Egger procedures were utilized in a sequential manner to identify and eliminate outliers with a *p*-value less than 0.05 (17). This iterative process was repeated until no outliers were detected.

### Statistical analysis

The statistical studies were conducted utilizing various packages in RStudio (version 4.3.1), including “Two Sample MR,” “MR-PRESSO,” “Phenosanner,” and “RadialMR.” In this study, an instrumental variable with an *F*-value >10 was considered a strongly correlated instrumental variable capable of reducing bias (18). The formula for calculating the *F*-value is as follows:


$$F=\left[\left(N-K-1\right)R^{2}\right]/\left[k\left(1-R^{2}\right)\right]$$

*R*^2^ is the fraction of variance that can be accounted for by genetic variation. *N* signifies the sample size, while *k* denotes the number of single nucleotide polymorphisms that have been incorporated.


$$R^{2}=\overset{k}{\underset{1}{\sum }}2\beta^{2}\left(1-EAF\right)EAF$$

EAF denotes the influence of the minor allele frequency, while *β* is utilized to signify the estimated effect on telomere length (19). The primary method employed for MR analysis was inverse variance-weighted regression, under the assumption of no instrumental variable invalidity. The *Q*-test was performed in order to evaluate the presence of heterogeneity among various genetic variants. In the context of heterogeneity, we utilized the MR-PRESSO method to identify and eliminate outliers in order to address the issue of horizontal pleiotropy. The study employed MR-Egger regression analysis to evaluate the presence of directional pleiotropy in the instrumental variables and to examine potential violations of the MR assumption. Furthermore, a leave-one-out sensitivity analysis was performed in order to evaluate the robustness of the results obtained from the MR analysis (20). Statistical significance was deemed to be present when the *p*-value was less than 0.05.

## Results

### The genetic correlation between telomere length and intracranial aneurysms

The genetic association between telomere length and intracranial aneurysms was evaluated using LD score regression, as presented in Table 2. There was an observed inverse relationship between telomere length and the occurrence of cerebral aneurysms (*R*_g_ = −0.13, *p* = 0.001).

**Table 2:** Genetic correlation estimates from LDSC regression between TL and IA.

Exposure	Outcome	*R*_g_	*R*_g__SE	*p*-value
TL	IA	−0.13	0.041	0.001

LDSC, linkage disequilibrium score; *R*_g_, genetic correlation; *R*_g__SE, the standard error of *R*_g_.

### The results of forward Mendelian randomization analysis

This study was undertaken employing MR analysis to examine the causal association between telomere length and intracranial aneurysms. The findings of the study are shown in the Table 3. The IVW analysis revealed a statistically significant association between telomere length and intracranial aneurysms. The IVW estimate (OR = 0.66, 95% CI: 0.47–0.92, *p* = 1.49 × 10^−2^) implied that greater telomere length was linked to a decreased chance of developing intracranial aneurysms.

**Table 3:** Mendelian randomization analysis of the association between risk of IA and TL.

Exposure	Outcome	Methods	NSNPs	Beta	SE	*p*-value	OR (95% CI)
TL	IA	MR Egger	47	−0.352	0.409	0.394	0.703 (0.316–1.566)
TL	IA	Weighted median	47	−0.508	0.258	0.049	0.602 (0.363–0.998)
TL	IA	Inverse variance weighted	47	−0.421	0.173	0.015	0.656 (0.467–0.921)
TL	IA	Simple mode	47	−0.666	0.473	0.166	0.514 (0.203–1.300)
TL	IA	Weighted mode	47	−0.558	0.319	0.088	0.573 (0.306–1.071)

NSNPs, number of single-nucleotide polymorphisms; OR, odds ratio; CI, confidence interval.

**Figure 1:**
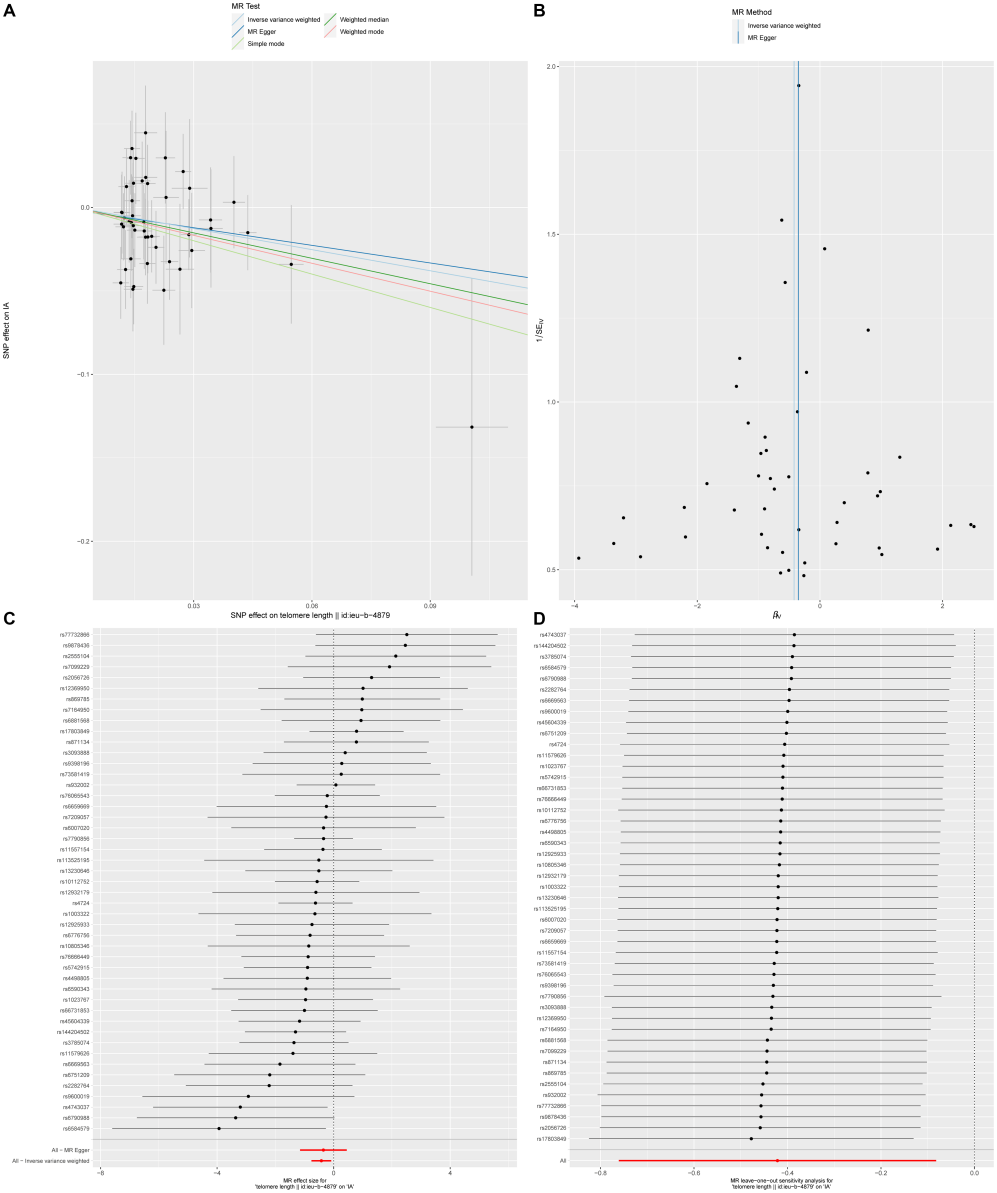
The causal impact of telomere length on intracranial aneurysms **(A)** Scatterplot illustrating the association between telomere length and intracranial aneurysms. **(B)** Funnel plot assessing the presence of heterogeneity. **(C)** Forest plot of SNPs related to both telomere length and intracranial aneurysms. **(D)** Leave-one-out sensitivity analysis evaluating the influence of each SNP in the causal relationship.

In order to conduct a more comprehensive investigation of the correlation between telomere length and intracranial aneurysms, we conducted studies to assess heterogeneity, sensitivity, and multiple effects. The findings of the study indicate that there was no statistically significant evidence of horizontal pleiotropy in the relationship between telomere length and intracranial aneurysms (Egger intercept = −0.002, *p* = 0.852). Additionally, no significant heterogeneity was observed in the impact of telomere length on intracranial aneurysms across different instrumental variables (Cochran *Q* = 40.287, *p* = 0.672) (Table 4). In a similar vein, the funnel plots of the IVW and MR Egger methods in Figure 1 did not exhibit any noteworthy heterogeneity among instrumental variables. The findings of sensitivity analysis conducted using the leave-one-out strategy indicated that the exclusion of any individual single nucleotide polymorphism out of the 47 SNPs related with intracranial aneurysms did not yield statistically significant changes in the outcomes. This implies the dependability of the results obtained from the MR analysis.

**Table 4:** Heterogeneity and horizontal pleiotropy of the associations between TL and the risk of IA.

Exposure	Outcome	Pleiotropy test			Heterogeneity test					
		MR-Egger			MR-Egger			Inverse-variance weighted		
		Intercept	SE	*p*-value	*Q*	*Q*_df	*Q*_pval	*Q*	*Q*_df	*Q*_pval
TL	IA	−0.002	0.008	0.852	40.287	45	0.672	40.322	46	0.708

SE, standard error.

### The results of reverse Mendelian randomization analysis

Reverse MR analysis implies that there is no causal relationship between intracranial aneurysm (IVW: OR = 1.008, 95% CI: 0.998–1.018, *p* = 0.11) and telomere length, as shown in Table 5 and the Supplementary Figure S2. The forest plot illustrates the causal effects of each SNP related to intracranial aneurysm on telomere length, as shown in the Supplementary Figure S2. The results of the reverse MR analysis imply that there is insufficient evidence to establish a causal association between intracranial aneurysm and telomere length. This conclusion is supported by the IVW method (IVW: OR = 1.008, 95% CI: 0.998–1.018, *p* = 0.11), as presented in Table 5 and the Supplementary Figure S2. The forest plot presented in the Supplementary Figure S2 depicts the causal effects of each SNP associated with intracranial aneurysm on telomere length.

**Table 5:** Mendelian randomization analysis of the association between TL and the risk of IA.

Exposure	Outcome	Methods	NSNPs	Beta	SE	*p*-value	OR (95% CI)
IA	TL	MR Egger	7	0.018	0.025	0.505	1.018 (0.970–1.069)
IA	TL	Weighted median	7	0.007	0.006	0.226	1.007 (0.996–1.019)
IA	TL	Inverse variance weighted	7	0.008	0.005	0.11	1.008 (0.998–1.018)
IA	TL	Simple mode	7	0.007	0.009	0.477	1.007 (0.989–1.025)
IA	TL	Weighted mode	7	0.008	0.008	0.4	1.008 (0.991–1.024)

NSNPs, number of single-nucleotide polymorphisms; OR, odds ratio; CI, confidence interval.

## Discussion

The present work utilized European GWAS data to conduct a two-sample bidirectional MR analysis. The primary objective was to examine the causal association between telomere length and the occurrence of intracranial aneurysms. The methodologies indicated above were used in this study’s forward MR analysis, which led to the selection of 49 SNPs, as shown in Supplementary Table S2. The results of leave-one-out sensitivity testing are shown in Supplementary Figure S1, where two outliers are found. After deleting these two outliers, the analysis was carried out once more using the identical procedures as earlier. The study employed genetic variations as surrogates for telomere length, and the results of the MR analysis implied a positive correlation between reduced telomere length and heightened susceptibility to intracranial aneurysms. The findings of the reverse MR analysis implied that there is no significant association between intracranial aneurysms and telomere length.

At present, investigations pertaining to the correlation between telomere length and intracranial aneurysms predominantly rely on animal experimentation. Fu et al. (2) utilized an animal model of intracranial aneurysms to demonstrate that the formation of intracranial aneurysms may be linked to increased expression of telomere-binding proteins, inhibition of telomerase enzyme activity, and telomere shortening. There exists a potential association between intracranial aneurysms and other artery aneurysms, as evidenced by instances of concurrent manifestation. This observation implies the plausibility of shared genetic and pathological factors contributing to the formation of these aneurysms (21). In their study, Shin et al. (3) performed a retrospective investigation on a cohort of patients presenting with both cerebral and arterial aneurysms. The authors observed common pathogenic mechanisms that were shared between these two types of aneurysms. The occurrence of aneurysms in the anterior circulation arteries after the bifurcation of the internal carotid artery, as well as ascending aortic aneurysms, may be influenced to a greater extent by genetic factors. The MR study conducted by Zhang et al. (4) provides additional evidence in support of a causal association between telomere length and arterial aneurysms. Specifically, the study found that individuals with longer telomere length had a decreased chance of developing arterial aneurysms. The findings of our study align with the results obtained from animal models of cerebral aneurysms and MR studies investigating the relationship between telomere length and arterial aneurysms.

The precise elucidation of the involvement of telomeres in the pathogenesis of aneurysms remains uncertain. Telomeres undergo a progressive shortening process during cellular division, and at reaching a critically short length, they induce cells to enter a state of senescence. The process of cellular senescence has the ability to induce harm to vascular tissues, resulting in the release of various inflammatory agents and the manifestation of the SASP (6). Numerous studies have provided evidence suggesting that inflammation has a substantial role in the initiation and progression of intracranial aneurysms (22–24). One viewpoint posits that the initiation of hemodynamic injury serves as a catalyst for a multifaceted inflammatory cascade that encompasses MMPs, vascular smooth muscle cells, macrophages, and oxidative stress. The genesis of intracranial aneurysms is believed to commence with endothelial dysfunction, which is triggered by oxidative stress. Following this, macrophages, mast cells, and many other inflammatory cells collaborate to initiate an inflammatory response. Macrophages play a crucial role in this process by producing and releasing MMPs, which are responsible for breaking down the extracellular matrix and collagen present in the artery wall. This mechanism amplifies the recruitment of inflammatory cells, increases the production of other proteases, and induces arterial wall remodeling and weakening, ultimately leading to the development and expansion of aneurysms. In addition, persistent inflammation can have a significant impact on the artery wall, resulting in the rupture of an aneurysm and the occurrence of subarachnoid haemorrhage (1, 25). Studies by Ali and colleagues have shown that tumor necrosis factor is involved in regulating the phenotypic switch of VSMCs towards a pro-inflammatory and matrix-remodeling phenotype (26). Research by Guo and colleagues has demonstrated a significant reduction in VSMC density in the medial layer of intracranial aneurysms compared to the normal arterial wall (27). These studies collectively suggest that inflammation is one of the primary factors contributing to the development of intracranial aneurysms. Therefore, the process of telomere shortening, which results in cellular senescence and the subsequent release of the SASP, has the potential to trigger an inflammatory reaction within the vascular wall. This mechanism could potentially contribute to the development of intracranial aneurysms.

Our present study possesses some notable strengths. The study employs publicly accessible large-scale GWAS data, consisting of 472,174 individuals, along with the aneurysm dataset from the International Stroke Genetics Consortium, which includes 7,495 cases and 71,934 controls. Furthermore, the employment of two-sample MR and diverse MR analysis techniques serves to alleviate the influence of confounding variables and address the issue of reverse causation. In conclusion, the present study evaluates the durability of the results by employing heterogeneity tests, sensitivity analysis, and various other methodologies.

Our research is subject to many constraints. Firstly, it is crucial to acknowledge that the sample consists exclusively of individuals of European ancestry. Therefore, the application of the study findings to non-European populations is uncertain, and additional confirmation is necessary for other groups of individuals. Then, it is significant that Mendelian randomization analysis does not include samples that overlap. The study has a maximum overlap rate of only 2% between the exposure and outcome datasets. To minimize potential bias, strong instrumental variables with high *F*-values are used. Furthermore, the use of disease-associated SNPs is a fundamental aspect of MR analysis. However, it is imperative to conduct further validation through the incorporation of other genetic association studies. Finally, our research implies a potential causal connection between telomere length and intracranial aneurysms. Nevertheless, the precise mechanisms that explain this connection are not yet understood, thus requiring additional investigation to explore its biological processes.

## Conclusion

In summary, this study used bidirectional two-sample Mendelian randomization analysis to imply a potential negative correlation between telomere length and intracranial aneurysms in the European population. It is necessary to validate these results in cohorts of different ethnicities and also to further investigate the underlying mechanisms.

## Data availability statement

The original contributions presented in the study are included in the article/Supplementary material, further inquiries can be directed to the corresponding author.

## Ethics statement

Ethical review and approval was not required for the study on human participants in accordance with the local legislation and institutional requirements. Written informed consent from the patients/participants or patients/participants' legal guardian/next of kin was not required to participate in this study in accordance with the national legislation and the institutional requirements.

## Author contributions

BX: Writing – original draft, Writing – review & editing. JR: Writing – original draft, Writing – review & editing. SZ: Writing – review & editing. YD: Writing – review & editing. WZ: Writing – review & editing. QG: Writing – review & editing. YF: Writing – review & editing. JZ: Writing – review & editing.
