# Supplementary material for: Causal relationship between telomere length and risk of intracranial aneurysm: a bidirectional Mendelian randomization study
Source: Front Neurol. 2024 Mar 11;15:1355895. doi: 10.3389/fneur.2024.1355895 (PMC10964484; doi:10.3389/fneur.2024.1355895)
Supplement: Supplementary file 1 [file Data_Sheet_1.PDF]

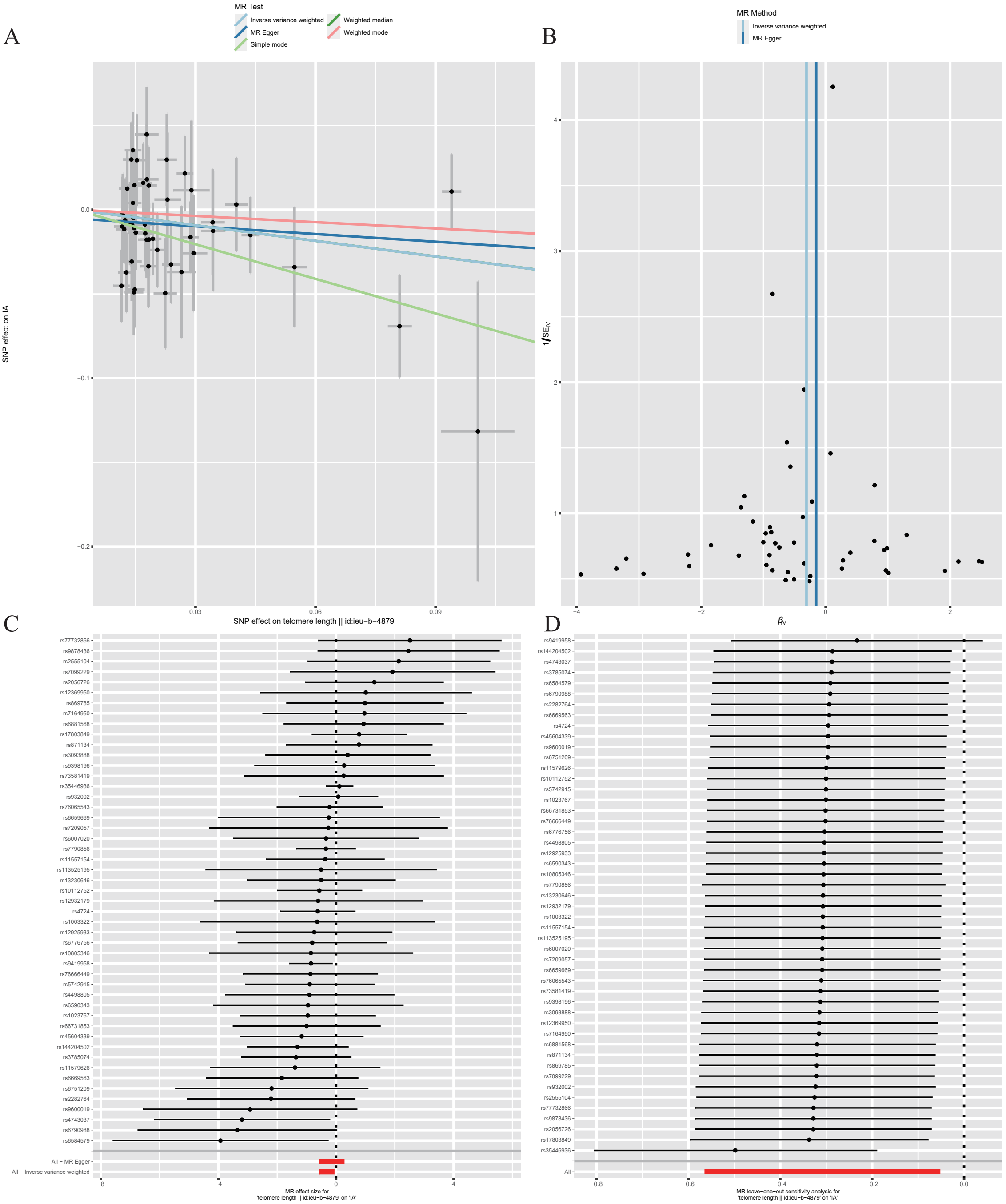

Supplementary Figure 1. The causal impact of telomere length on intracranial aneurysms (A) Scatterplot illustrating the association between telomere length and intracranial aneurysms. (B) Funnel plot assessing the presence of heterogeneity. (C) Forest plot of SNPs related to both telomere length and intracranial aneurysms. (D) Leave-one-out sensitivity analysis evaluating the influence of each SNP in the causal relationship.
