# Supplementary material for: Causal relationship between telomere length and risk of intracranial aneurysm: a bidirectional Mendelian randomization study
Source: Front Neurol. 2024 Mar 11;15:1355895. doi: 10.3389/fneur.2024.1355895 (PMC10964484; doi:10.3389/fneur.2024.1355895)
Supplement: Supplementary file 2 [file Data_Sheet_2.PDF]

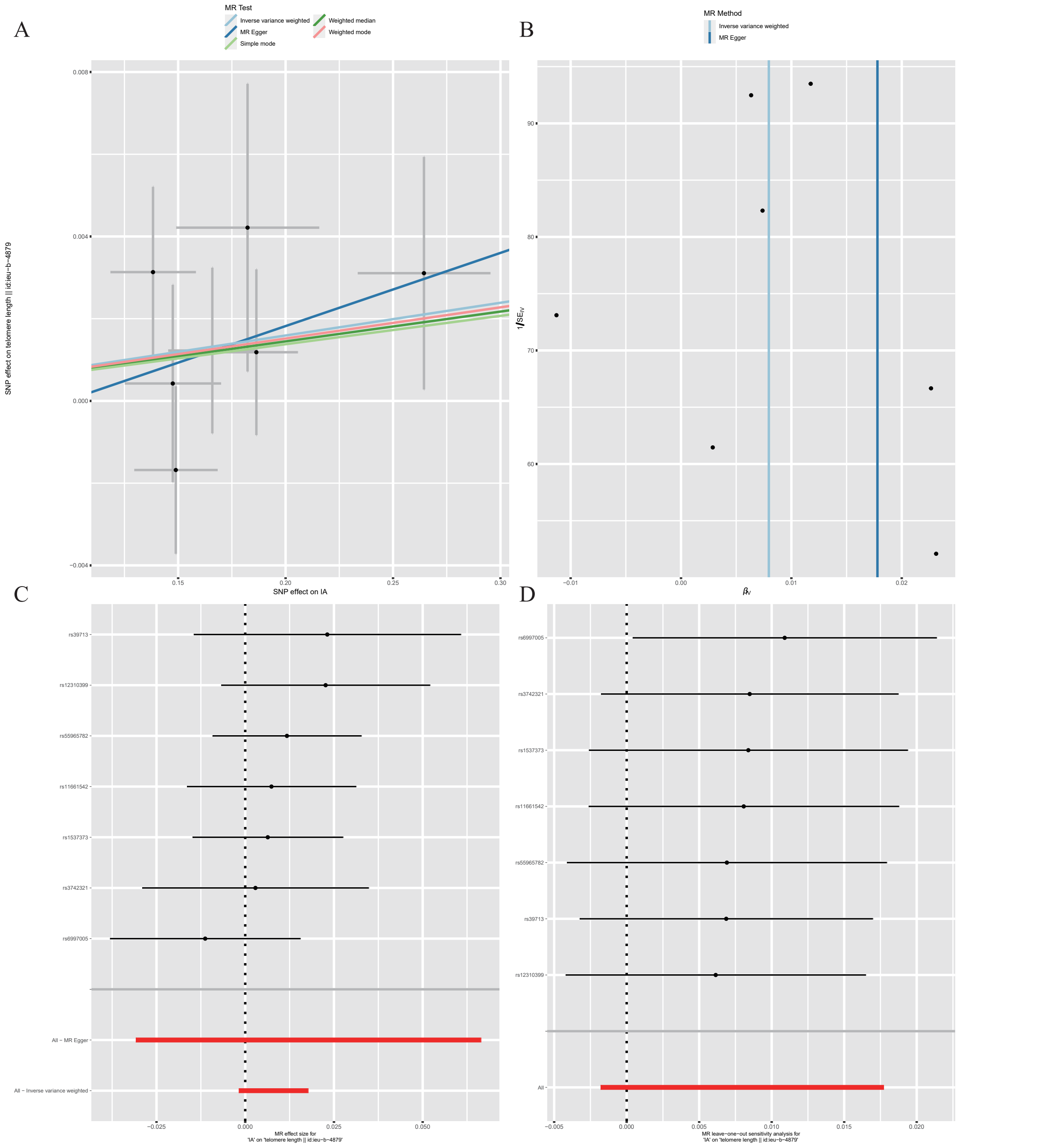

Supplementary Figure 2. The causal impact of intracranial aneurysms on telomere length (A) Scatterplot illustrating the association between intracranial aneurysms and telomere length. (B) Funnel plot assessing the presence of heterogeneity. (C) Forest plot of SNPs related to both intracranial aneurysms and telomere length. (D) Leave-one-out sensitivity analysis evaluating the influence of each SNP in the causal relationship.
